# The Chapel of Guy's Hospital

**Published:** 1917-06-30

**Authors:** 


### THE EDITOR'S LETTER-BOX.

### The Chapel of Guy's Hospital.

*To the Editor of* THE HOSPITAL.

SIR,—All will be glad that in the Hospital Sunday Number of your paper you should have emphasised the work of our hospital chapels; but would it not have been more useful if your article on "The Chapel of Guy's Hospital" had been written by one who knew a little of the considerable work of the chaplains and the spirit in which that work is undertaken? Nothing is said of the daily ministrations of the chaplains in the wards, of their readiness to take the Holy Communion in the early morning to those who desire it or at any hour to the dying, of the ward services throughout the week, of their work for the hospital staff, and any stranger to the hospital might be forgiven if he supposed after reading the article that the chaplains received "£350 per annum" between them to read the lessons "indistinctly" (the only mention of their work), and that the really important spiritual work for the patients was undertaken between "5.30 P.M. and 7 P.M." on a Sunday by the "Sunday preachers"! The voluntary work of the latter is most generously given, because two chaplains cannot possibly serve all the many wards on Sunday, and means much self-denial; but to write, as your contributor has written, is comparable to saying that Belgium or Serbia or one of the South American Republics has undertaken to break the military power of Germany and Austria! It would have been better if the article had been written by some Guy's resident, or at least have been submitted to one of the chaplains before publication.—Yours, etc.,

ONCE A HOSPITAL CHAPLAIN.

*** We have pleasure in publishing this letter by a former chaplain of Guy's Hospital. The immediate cause of complaint deals with matters not within the ground covered by the article, which is, The Hospital Chapel and Sunday Services at Guy's. The daily administration of the chaplain within the wards could not be adequately dealt with within the space available, nor did it come within the immediate subject of the special article referred to. We may add, however, that we wrote to the chaplain, seeking information, and asking him to write an article, hoping in this way to best secure the desired end. We found, unfortunately, he was away. On his return, we hope to publish a further short article on the spiritual work at Guy's. This should advance the object we have had always in view, which is to secure in every hospital the fullest facilities for meeting the spiritual needs of the patients and the staff.—ED. THE HOSPITAL.

### The Royal Infirmary and our Prisoners in Germany: An Example which might be Followed.

*To the Editor of* THE HOSPITAL.

